# Whole Organism Model to Study Molecular Mechanisms of Differentiation and Dedifferentiation †

**DOI:** 10.3390/biology9040079

**Published:** 2020-04-17

**Authors:** Areeba Anwar, Ruqaiyyah Siddiqui, Naveed Ahmed Khan

**Affiliations:** 1 Department of Biological Sciences, School of Science and Technology, Sunway University, Bandar Sunway 47500, Malaysia; areeba_anwar@ymail.com; 2 Department of Biology, Chemistry and Environmental Sciences, College of Arts and Sciences, American University of Sharjah, University City 26666, UAE; rsiddiqui@aus.edu

**Keywords:** cell differentiation, cancer, acanthamoeba, phenotypic switching, encystation, protozoa, cancer stem cells, dedifferentiation, signalling, apoptosis

## Abstract

Cancer recurrence has remained a significant challenge, despite advances in therapeutic approaches. In part, this is due to our incomplete understanding of the biology of cancer stem cells and the underlying molecular mechanisms. The phenomenon of differentiation and dedifferentiation (phenotypic switching) is not only unique to stem cells but it is also observed in several other organisms, as well as evolutionary-related microbes. Here, we propose the use of a primitive eukaryotic unicellular organism, *Acanthamoeba castellanii*, as a model to study the molecular mechanisms of cellular differentiation and dedifferentiation.

## 1. Introduction

Current therapeutic approaches in the treatment of cancer prolong patients’ survival rates but successful prognosis in the case of metastasis and tumour recurrence remains inadequate [1]. In part, this is due to cancer stem cells (CSCs) which can remain dormant for years, displaying minimal metabolic activity and thus escape treatment. Once favourable conditions return, CSCs have the ability to self-renew, differentiate and dedifferentiate, invade, and metastize [2], albeit it is not clear what conditions are considered favourable to CSCs. Hence, understanding the biology of CSCs and the underlying molecular mechanisms is an important strategy to develop novel therapeutic methods [3,4,5]. The phenomenon of differentiation and dedifferentiation (phenotypic switching) is not unique to CSCs and is observed in several other organisms as well as evolutionary-related microbes. In fact, phenotypic switching is prevalent throughout all the subdomains of eukaryotes. These include Holozoa (e.g., *Choanoflagellates*), Fungi (e.g., *Fonticula alba*), Amoebozoa (e.g., *Acanthamoeba*), Alveolata (e.g., *Dinoflagellates*), Rhizaria (e.g., *Cercozoa*), Stramenopila (e.g., *Haptophytes*), Viridiplantae (e.g., *Prasinophytes*), Excavata (e.g., *Euglena*), and Jakobida (e.g., *Reclimonas*) [6]. Regardless of the widespread presence of the phenomenon of phenotypic switching among eukaryotes, the molecular details and the precise underlying mechanisms are still insufficient [6].

Dormancy is a quiescent time period of a cell in which it remains arrested in the G0/G1 phase. Dormant cells show no sign of proliferation but retain their malignant properties [7,8]. It is hypothesized that 20% of breast cancer patients who had undergone surgery and had been clinically declared cancer free showed recurrence after 5–25 years due to the re-activation of CSCs [9,10,11,12].

CSCs were first identified in acute myeloid leukemia (a tumor of hematopoietic origin) [3,4]. CSCs have the ability to invade, metastasize, and divide asymmetrically into an identical daughter cell [13,14]. Studies report that disseminated tumor cells (DTCs) and circulating tumour cells (CTCs) might disperse early during the process of primary tumorigenesis [12,15,16]. An incomplete understanding of CSC characterization, specific identification, uncertainty of biomarkers, isolation, culturing of CSCs in vitro, and transition of CSCs into dormant and active malignant states contributes to challenges in developing therapeutics [17,18,19]. 

Here, we propose the use of the primitive eukaryotic unicellular organism *Acanthamoeba castellanii* as a model to study the molecular mechanisms of cellular differentiation and dedifferentiation. *Acanthamoeba castellanii* is an ancestral eukaryotic and a unicellular organism [20]. *Acanthamoeba* has already been used as a model to study different metabolic, proliferative, and survival mechanisms in eukaryotic organisms [21,22,23]. They exhibit an invasive strategy, i.e., capture their target by actin-dependent phagocytosis, and can switch their phenotypes according to the diverse environmental conditions. This ability of phenotypic switching and survival in adverse environment renders *Acanthamoeba* an attractive model to study cellular processes at the molecular level and thus unveils the molecular mechanisms of differentiation and dedifferentiation. 

## 2. Perspective: Similarities and Dissimilarities between CSCs and *Acanthamoeba*

### 2.1. Genomics and Proteomics

#### 2.1.1. Differentiation and Dedifferentiation

Embryonic stem cells have the ability to self-renew infinitely in culture. They are pluripotent in nature and can differentiate into all types of cell lineages. A study carried out by Takahashi and Yamanaka showed that, in the adult mouse fibroblast model, insertion of four transcription factors, that is, Oct-3/4, Sox2, c-Myc, and KLF4 resulted in the generation of cell colonies having properties of ESCs [23]. When transplanted in immunodeficient mice, these colonies were able to differentiate into endodermal, ectodermal, and mesodermal cell types and were then termed as induced pluripotent cells (iPSCs). Various other methods have also been developed of the generation of iPSCs [24,25].

The term phenotypic heterogeneity is used for various functional properties and the expression of different biomarkers a tumour cell can possess during tumorigenesis and metastasis. Subpopulations of the tumour cells can be identified based on the presence of cell surface markers indicating the presence of various states of cellular differentiation [26].

A number of studies provide evidence of the early stated hypothesis of bidirectional differentiation or dedifferentiation from a non-CSC to CSC, also termed *tumour cell plasticity*. CSCs not only have the ability to differentiate and dedifferentiate but also, as discovered in glioblastoma, can transdifferentiate to endothelial cells, leading to angiogenesis [27,28]. Intestinal cancers also have bidirectional conversion of CSCs and non-CSCs. A study was carried out which showed that, by the activation of NF-κB, the Wnt signalling pathway was upregulated, leading to the reprogramming in inflammatory stroma, that is dedifferentiation [14]. A study reported that oncogenic mutation of Kras and TNF-α-dependent NF-κB signalling initiation leads to the activation of Wnt/β-catenin/Tcf pathway which leads to dedifferentiation of non-SIEC (non-stem intestinal epithelial cells) into neoplastic cells, which is in contrast to the unidirectional model of differentiation pattern of stem cells [29].

This bidirectional conversion of active and inactive cell forms can similarly be seen in the transition of an *Acanthamoeba* trophozoite into the dormant cyst form, which involves a number of signalling mechanisms. Although these mechanisms are not fully understood, studies report that expression of cyst encoding genes and proteins is upregulated, functionally similar to the Wnt/β-catenin pathway. These proteins include proteins of the cellulose synthesis pathway [30], cyst wall proteins like CSP2 [31], and polyphenol oxidase [32]. CSP21 is not detectable in trophozoites but can be detected after 12 h of differentiation. A study reported that CSP21 gene expression is active when its specific repressor molecule is removed. This repressor could be a DNA-binding protein like TBPF, studied previously in *Acanthamoeba* [33,34]. During differentiation, certain genes of large rRNA, 5S rRNA, and of ribosomal protein [35,36] are downregulated. However, the transcriptional activity of TBP (TATA box-binding protein) and its promoter binding factor (TBPF), RNA polymerase II, remain unaffected during differentiation. Likewise, the expression of other proteins such as the protein disulphide isomerase and cytoskeletal proteins (tubulin, myosin, actin, extendin, and ubiquitin) also remain constant [37,38]. When the inhibitor Rho kinase (Y27632, small GTPase), a regulator of actin polymerization, was tested, encystment of *Acanthamoeba* was blocked [39]. This indicates that the process of cytoskeletal rearrangement is involved while there is conversion of trophozoite into cyst. The proteases family involved in pathogenesis of cancer include matrix-metallo, serine, cysteine, threonine, and aspartic proteases, having pro- and antitumour functions [40]. A study by Gopinath et al. (2013) demonstrated the elevated expression of cysteine protease (cathepsin B alone or with uPAR) in glioblastomas, which in turn was responsible of self-renewal of malignant glioblastoma stem cells. This was regulated by the hedgehog pathway (Gli2, Bmi1, and Sox 2) to promote tumour initiation and maintenance [41].

In *Acanthamoeba*, only the transcription of profilin and two CDKs is found to be upregulated during initial 16 h of transition phase [38]. The other genes involved in mediation and regulation of encystation include protein kinase C, that is homologically similar to xylose isomerase; Na P-type ATPase, heat shock protein (HSP), cullin 4, and autophage protein 8 have been identified in *Acanthamoeba* [42,43,44]. The expression of subtilisin-like serine protease and cysteine protease is also induced when encystation begins. This is due to the requirement of protein turnover, which is carried out by lysosomal and ubiquitin dependent proteases [43,44,45].

The levels of adenylate cyclase activity rises 2–4-fold during dormant stage [46]. The cAMP levels also increase initially during differentiation but then get back to normal levels observed in the growth phase. Cyclic AMP exhibits its mechanism via protein kinase mediated system. This affects different levels such as transcription, translation, and posttranslational modifications [47]. Another signalling mechanism involves high expression of PKC-like genes (21 types) during the process of encystation [48]. Mortazavi et al. (2010) have shown the activities of phospholipase A2 in *Acanthamoeba* cultures [49], whereas in CSCs, the knockdown of secretory phospholipase A2, similar to *Acanthamoeba*, were shown to inhibit cancer stem cells phenotype in vitro [50].

#### 2.1.2. Signalling Pathways

The Ras family in humans have important roles in signalling cascades including proliferation, metabolism, cell adhesion and survival, differentiation, and maintenance of cytoskeleton integrity [51]. Elevated expression of p38 (cell cycle regulator)-MAPK signalling-induced cell cycle arrest in in vivo models of breast cancer, prostate cancer, melanoma, and fibrosarcoma results in tumour dormancy [7,52]. PTEN mutation in cancers induces constitutive expression of PI3K signalling effector molecules [53].

The presence of very few signalling pathways have been identified in *Acanthamoeba*. Evidence of the Ras pathway was obtained by the inhibition of enzyme farnesyl protein transferase (FPT III). FPT III inhibits Ras farnesylation, which led to the reduced levels of encystation [39]. Inhibitors of MAPK, which is a subsequent protein activated by Ras signalling, did not show any effects on the process of differentiation. The MAPK pathway has been found to be associated with encystment process by homologues proteins of ErkA and ErkB [54]. Ras activation could also be involved with the tyrosine kinase receptor family (RTK), since in a study conducted on another type of free-living amoeba *Balamuthia*, inhibitors of PI3K and RTK inhibitors decreased the rate of encystation [55]. However, different triggering molecules may be involved in activating the different signalling mechanisms.

Impaired Notch and Hedgehog signalling are responsible for stem cell hierarchy and differentiation. Both of these pathways, which are found deregulated in different types of cancers including breast, prostrate, colorectal, lung, CNS malignancies, and T-cell leukemia [56,57], have not been identified in *Acanthamoeba* yet.

#### 2.1.3. Cell Cycle

The cell cycle is an integral part of cellular processes. The transition of one phase to the other in the G_o_/G1, S, and G2/M phases of the cell cycle in cancer cells occurs only after passing through the checkpoints, regulated by cyclins and CDKs, which is impaired in cancer. It is reported that dormant cancer cells remain in the G_o_/G1 phase of the cell cycle. One of the main checkpoint modulator of the cell cycle, p38, has been found to be greatly associated with dormant phase in several tumour types [58].

However, in the case of *Acanthamoeba*, it was reported that trophozoites are formed in the mitotic phase of cell division cycle. Moreover, the absence of the G1 phase has been observed in some protists including *Amoeba proteus* [59]. However, an extended G1 phase can be observed under certain conditions. In most of the cases, the G2 phase is more than 50% of the total cell cycle period. Different studies report that cells in the late G2 phase undergo the process of differentiation into cysts when faced with harsh environmental conditions [60,61,62,63]. It is interesting to study the initiation and regulation of differentiation in cells having no G1 phase, as typically, cell differentiation occurs from the G1 phase of the cell cycle. Mengue et al. (2016) have reported the presence of functional CDK, CDC2b, in *Acanthamoeba*, which regulated cell cycle progression upon its inhibition [64].

#### 2.1.4. Apoptosis

The transition between active phases of dormancy, apoptosis induction, and proliferation is strictly controlled. Apoptosis is evaded by cancer cells due to developed resistance. The three major ways which enable cancer cells to escape apoptotic pathways include (i) imbalanced ratio of proapoptotic and antiapoptotic protein function, (ii) decreased function of caspases, and (iii) fault in death receptor signaling [65].

Apoptosis is a tightly regulated mechanism which is evaded by cancer cells due to the developed resistance. The proteins of the Bcl-2 family are known to have proapoptotic (Bax and bid) or antiapoptotic/cell survival (Bcl-2 and Bcl-xL) activities [66]. Mutation in the P53 protein [67], decreased function of caspases, and faults in death receptor signalling due to the disturbed ratio of pro- and antiapoptotic proteins are the reason for impaired apoptosis [65].

Studies report that the morphological traits of intrinsic apoptosis have been observed in *Acanthamoeba*, including DNA fragmentation, detection of phosphatidylserine on the outer side of the cell membrane, cell size shrinkage, cellular permeability, and changes in mitochondrial membrane potential [68,69,70]. As mostly caspases are involved in inducing apoptosis [71], in parasites, metacaspase and caspase-1 (interleukin-1 converting enzyme (*ICE*)-like protease) have shown to function during apoptosis induction [72,73]. Recently, Nakisah et al. (2012) and Feng et al. (2009) have reported programmed cell death occurring in trophozoites of *Acanthamoeba* [74,75]. In 2018, Wu et al. have shown the induction of apoptosis in *Acanthamoeba* by oleic acid. They showed that apoptosis is triggered by activation of caspase 3 and upregulation of MCA *Atg3*, *Atg8*, LC3A/B protein, and caspase-1 [76]. The presence of similar mechanisms at the genomics and proteomic levels in *Acanthoamoeba*, as in CSCs, further supports our hypothesis.

## 3. Metabolomics

It is reported that glycolysis is elevated in CSCs in comparison to differentiated cancer cells. CSCs lead to the enhanced production of lactate due to the greater uptake of glucose. This is due to the upregulated levels of glycolytic enzymes (hexokinase II, Glut1, and lactate dehydrogenase A) and reduced metabolism in mitochondria. However, opposingly, it also reports that, in CSCs, oxidative metabolism is the main source of energy, but reactive oxygen species (ROS) levels remain lesser than in differentiated cancer cells [77].

*Acanthamoeba* also becomes metabolically inactive after the transition from trophozoite into the cyst stage. The reduction in RNA, proteins, fatty acids, and sugar levels occurs during the encystation, which results in dry weight and lesser cellular volume [78]. The evidence of the presence and regulation of different enzymes levels has been observed in the transition, such as isocitrate lyase, isocitrate dehydrogenase, glycolate, and maleate [79]. Upon inhibition of enzymes involved in polyamine biosynthesis, S-adenosyl- L-methionine decarboxylase has also shown a metabolic role in differentiation [80]. Another two enzymes of the glycolytic pathway, namely enolase and fructose bisphosphate aldolase, have also been found to play roles in cyst formation [81].

## 4. Environmental Factors

Interaction of tumour microenvironmental factors with CSCs helps in retaining their stem cell properties and in providing resistance against therapy [82]. Hypoxia also triggers the dedifferentiation of non-CSCs to CSCs [83], enrooting to malignancy [84]. Under the stress of hypoxic conditions, increased glycolysis is favoured in CSCs by transcription factor HIF-1, which increases the expression of glycolytic enzymes [83]. Immunosurveillance strategy present in CSCs is due to the presence of low levels of MHC-1 and β-macroglobulin and elevated levels of Bcl-2, Bcl-xL, and survivin, which helps CSCs to escape from cells of the immune system [85].

In the same way, to survive under harsh and extreme environmental conditions like CSCs, resistant and dormant forms of *Acanthamoeba* have been isolated from places, such as Antarctica [86], sediments of the ocean [87], and under deep sea water [88]. This indicates their ability to undergo respiration under low oxygen and at extreme pH levels [86]. Being a eukaryotic organism, *Acanthamoeba* undergoes the glycolytic pathway (conversion of glucose into pyruvate) and then oxidative phosphorylation in mitochondria [20]. Under hypoxic conditions, a hydrogenosomal-type anaerobic generation of ATP was described in *Acanthamoeba castellanii* [89], similar to the mechanism adapted by CSCs as mentioned above. During anaerobic conditions, pyruvate ferredoxin oxidoreductase catalyses the decarboxylation of pyruvate instead of typical pyruvate dehydrogenase [89]. This ability of survival in extreme conditions is similar to cancer cells which metastasize inside the body despite various environmental factors and given treatment. Achar and Weissman (1980) have confirmed that increased intracellular levels of cyclic AMP are attained when *Acanthamoeba* cells in the late log phase are transferred to an encystation medium [46]. However, upon the return of favourable growth conditions, amoebic cysts are transformed back into trophozoites, leading to reproduction and infection recurrence [90].

Conventional chemotherapy mostly targets the fast-growing neoplastic cells but not the hidden CSCs. Cancer stem cell biology has to be understood in detail to specifically identify the molecular drug targets unique to the CSCs. Differentiation therapy has also been proposed to treat malignant cancers. It targets the CSCs to differentiate into mature cancer cell type. The use of differentiation inducing agents or ligands to the tumour areas, positive or negative regulatory molecules in asymmetric mitotic signalling, various gene products, and anti-sense or ribozyme agents are the options to induce differentiation in CSCs [91]. However, tumour plasticity and existence of CSCs niches still remain a technical challenge in developing new treatment strategies [92].

In Table 1, we have depicted the inhibitors targeting cancer stem cells which are already in clinical trials [93]. The mentioned compounds studied for targeting CSCs can also be tested against *Acanthamoeba* for their effects on the process of differentiation and dedifferentiation, as currently there is no single method of treatment for *Acanthamoeba* infections due to resistant cysts forms and inability to cross the blood brain barrier in case of AK and AGE, respectively [94]. The compounds mentioned in Table 1 target different molecular pathways in humans, and genes similar to these are also identified in amoeba, as mentioned previously in the section *Genomics and Proteomics*. Therefore, this attempt will demonstrate the relevance of the suggested model to the cancer stem cells, and similarities in between the molecular biology of amoeba and cancer stem cells can be explored further.

Table 2 summarizes the comparisons between CSCs and the eukaryotic organism *Acanthamoeba* highlighting the use of the eukaryotic model organism in studying the processes of differentiation, molecular signalling, cell cycle, and apoptosis. The phenotypic resemblance between tumour dormancy and encystation, existence of comparable molecular features, and strategies of survival are evident of the presence of ancestral *“Mother”* features of eukaryotic cellular organism post-biological evolution. *Acanthamoeba* has already been used as a model to study cell motility comprehensively at the molecular level [95]. However, studying the specific genetic mutations, upregulated and downregulated signalling pathway of different cancer types can be a challenge. Once the basic model is developed and functioning, then different techniques can be used to induce targeted gene mutations. This has been demonstrated in a study by Sekine et al., in 2018, in which they used CRISPR/Cas9 technology to edit multiple genes in *Dictyostelium discoideum*, a eukaryotic amoebic study model [96]. Moreover, gene transfection methods and RNA interference can also be helpful in this regard [97].

The use of a realistic experimental model can help us determine the complex molecular mechanisms of dormancy and recurrence with the help of gene microarrays and advance techniques in metabolomics and proteomics [98]. This will enable us to fill the current gaps in understanding and to establish effective therapeutic modalities.

## Figures and Tables

**Table 1:** Inhibitors targeting cancer stem cells that can be potential drugs against *Acanthamoeba* differentiation [91,93].

Inhibitors	Target	Cancer Type	Phase
Vitamin D3 [91]	β-catenin	Basal Cell Carcinoma	III
PRI-724 [91]	CBP/β-catenin	advanced solid tumors	I
CWP232291 [91]	β-catenin	Acute myeloid leukemia (AML)	I
MK0752 [91]	γ-secretase	Advanced Breast Cancer	I
RO4929097 [91]	γ-secretase	Lung Cancer	II
PF-03084014 [91]	γ-secretase	Leukemia	I
OMP-21M18 [91]	anti-DLL4	Pancreatic Cancer	I
BMS-833923 [91]	Smoothened (SMO)	Basal cell	I
IPI-926 [91]	SMO	Primary Myelofibrosis Fibrosis and Bone Marrow	II
IPI-926 [91]	Hedgehog	Recurrent Head and Neck Cancer	I

**Table 2:** Comparative properties of cancer stem cells and *Acanthamoeba.*

Properties	Cancer Stem Cells (CSCs)	*Acanthamoeba*
*Genomics and Proteomics*		
Apoptosis	+ (Caspases, Bcl-2 family, and p53) [66,67]	+ Metacaspase, caspases 1 and 3, MCA *Atg3*, *Atg8*, and LC3A/B protein [72,73,76]
Cell Cycle	+ Cyclins, CDKs [58]	+ CDC2b [64]
Signalling Mechanisms		
*Ras Pathway*	+ [51]	+ [39]
*MAP Kinase Pathway*	+ [52]	+ [55]
*PI3K Pathway*	+ [52]	+ [55]
*Wnt/* *β catenin Pathway*	+ [14]	CSP21 [31]
*Hedgehog Pathway*	+ [57]	Unknown
*Notch Pathway*	+ [56]	Unknown
*Metabolomics*		
Glycolysis	+ ATP-dependent phosphofructokinase and all glycolytic enzymes [5]	+ Pi-dependent phosphofructokinase, isocitrate lyase, isocitrate dehydrogenase, glycolate, maleate, enolase, and fructose bisphosphate aldolase [79,80,81]
Phospholipases	+ [50]	+ [49]
Proteases	+ [40]	+ [45]
*Environmental factors*		
Glycolysis under hypoxic conditions/survival in stress	+ [83]	+ [89]
Dormancy/differentiation	+ Non-CSCs to CSCs [82]	+ (encystation) [20]
Growth under optimum conditions/dedifferentiation	+ Differentiation and malignancy [83]	+ Excystation and opportunistic infection [90]

## References

[1] Batlle E., Clevers H. (2017). Cancer stem cells revisited. Nat. Med..

[2] Ayob A.Z., Ramasamy T.S. (2018). Cancer stem cells as key drivers of tumour progression. J. Biomed. Sci..

[3] Bonnet D., Dick J.E. (1997). Human acute myeloid leukemia is organized as a hierarchy that originates from a primitive hematopoietic cell. Nat. Med..

[4] Lapidot T., Sirard C., Vormoor J., Murdoch B., Hoang T., Caceres-Cortes J., Dick J.E. (1994). A cell initiating human acute myeloid leukaemia after transplantation into SCID mice. Nature.

[5] De Francesco E.M., Sotgia F., Lisanti M.P. (2018). Cancer stem cells (CSCs): Metabolic strategies for their identification and eradication. Biochem. J..

[6] Schaap P., Schilde C. (2018). Encystation: The most prevalent and underinvestigated differentiation pathway of eukaryotes. Microbiol. J..

[7] Aguirre-Ghiso J.A. (2007). Models, mechanisms and clinical evidence for cancer dormancy. Nat. Rev. Can..

[8] Goss P.E., Chambers A.F. (2010). Does tumour dormancy offer a therapeutic target?. Nat. Rev. Can..

[9] Karrison T.G., Ferguson D.J., Meier P. (1999). Dormancy of mammary carcinoma after mastectomy. J. Nat. Can. Inst..

[10] Saphner T., Tormey D.C., Gray R. (1996). Annual hazard rates of recurrence for breast cancer after primary therapy. J. Clin. Oncol..

[11] Democheli R., Tereziani M., Valagussa P., Moliterni A., Zambetti M., Bonadonna G. (1994). Local recurrences following mastectomy: Support for the concept of tumor dormancy. J. Nat. Can. Inst..

[12] Meng S., Tripathy D., Frenkel E.P., Shete S., Naftalis E.Z., Huth J.F., Uhr J.W. (2004). Circulating tumor cells in patients with breast cancer dormancy. Clin. Can. Res..

[13] Sell S. (1993). Cellular origin of cancer: Dedifferentiation or stem cell maturation arrest?. Environ. Health Perspect..

[14] Friedmann-Morvinski D., Verma I.M. (2014). Dedifferentiation and reprogramming: Origins of cancer stem cells. EMBO Rep..

[15] Hüsemann Y., Geigl J.B., Schubert F., Musiani P., Meyer M., Burghart E., Forni G., Eils R., Fehm T., Riethmüller G. (2008). Systemic Spread Is an Early Step in Breast Cancer. Cancer Cell.

[16] Schmidt-Kittler O., Ragg T., Daskalakis A., Granzow M., Ahr A., Blankenstein T.J.F., Kaufmann M., Diebold J., Arnholdt H., Müller P. (2003). From latent disseminated cells to overt metastasis: Genetic analysis of systemic breast cancer progression. Proc. Natl. Acad. Sci. USA.

[17] Pantel K., Brakenhoff R.H. (2004). Dissecting the metastatic cascade. Nat. Rev. Cancer.

[18] Pantel K., Brakenhoff R.H., Brandt B. (2008). Detection, clinical relevance and specific biological properties of disseminating tumour cells. Nat. Rev. Cancer.

[19] Kemper K., Sprick M.R., de Bree M., Scopelliti A., Vermeulen L., Hoek M., Zeilstra J., Pals S.T., Mehmet H., Stassi G. (2010). The AC133 epitope, but not the CD133 protein, is lost upon cancer stem cell differentiation. Cancer Res..

[20] Khan N.A. (2006). *Acanthamoeba*: Biology and increasing importance in human health. FEMS Microbiol. Rev..

[21] Riyahi T.Y., Frese F., Steinert M., Omosigho N.N., Glöckner G., Eichinger L., Orabi B., Williams R.S., Noegel A.A. (2011). RpkA, a highly conserved GPCR with a lipid kinase domain, has a role in phagocytosis and anti-bacterial defense. PLoS ONE.

[22] Baig A.M., Ahmad H.R. (2017). Evidence of a M1-muscarinic GPCR homolog in unicellular eukaryotes: Featuring *Acanthamoeba* spp bioinformatics 3D-modelling and experimentations. J. Recept. Signal Transduct..

[23] Takahashi K., Yamanaka S. (2006). Induction of Pluripotent Stem Cells from Mouse Embryonic and Adult Fibroblast Cultures by Defined Factors. Cell.

[24] Sommer C.A., Mostoslavsky G. (2013). The evolving field of induced pluripotency: Recent progress and future challenges. J. Cell. Physiol..

[25] Malik N., Rao M.S. (2013). A review of the methods for human iPSC derivation. Methods Mol. Biol..

[26] Visvader J.E., Lindeman G.J. (2012). Cancer stem cells: Current status and evolving complexities. Cell Stem cell..

[27] Soda Y., Marumoto T., Friedmann-Morvinski D., Soda M., Liu F., Michiue H., Pastorino S., Yang M., Hoffman R.M., Kesari S. (2011). Transdifferentiation of glioblastoma cells into vascular endothelial cells. Proc. Natl. Acad. Sci. USA.

[28] Ricci-Vitiani L., Pallini R., Biffoni M., Todaro M., Invernici G., Cenci T., Maira G., Parati E.A., Stassi G., Larocca L.M. (2010). Tumour vascularization via endothelial differentiation of glioblastoma stem-like cells. Nature.

[29] Schwitalla S., Fingerle A.A., Cammareri P., Nebelsiek T., Goktuna S.I., Ziegler P.K., Canli O., Heijmans J., Huels D.J., Moreaux G. (2013). Intestinal tumorigenesis initiated by dedifferentiation and acquisition of stem—cell-like properties. Cell.

[30] Potter J.L., Weisma R.A. (1972). Correlation of cellulose synthesis in vivo and in vitro during the encystment of *Acanthamoeba*. Dev. Biol..

[31] Hirukawa Y., Nakato H., Izumi S., Tsuruhara T., Tomino S. (1998). Structure and expression of a cyst specific protein of *Acanthamoeba castellanii*. Biochim. Biophys. Acta.

[32] Sykes D.E., Band R.N. (1985). Polyphenol oxidase produced during encystation of *Acanthamoeba castellanii*. J. Protozool..

[33] Gaston K., Jayaraman P.S. (2003). Transcriptional repression in eukaryotes: Repressors and repression mechanisms. Cell. Mol. Life Sci..

[34] Huang W., Bateman E. (1997). Transcription of the *Acanthamoeba* TATA-binding Protein Gene a single transcription factor acts both as an activator and a repressor. J. Biol. Chem..

[35] Matthews J.L., Zwick M.G., Paule M.R. (1995). Coordinate regulation of ribosomal component synthesis in *Acanthamoeba castellanii*: 5S RNA transcription is down regulated during encystment by alteration of TFIIIA activity. Mol. Cell. Biol..

[36] Stevens A.R., Pachler P.F. (1973). RNA synthesis and turnover during density-inhibited growth and encystment of *Acanthamoeba castellanii*. J. Cell Biol..

[37] Detke S., Paule M.R. (1975). DNA-dependent RNA polymerases from *Acanthamoeba castellanii*: Properties and levels of activity during encystment. Biochim. Biophys. Acta.

[38] Orfeo T., Bateman E. (1998). Transcription by RNA polymerase II during *Acanthamoeba* differentiation. Biochim. Biophys. Acta.

[39] Dudley R., Jarroll E.L., Khan N.A. (2009). Carbohydrate analysis of *Acanthamoeba castellanii*. Exp. Parasitol..

[40] Hillebrand L.E., Reinheckel T. (2019). Impact of proteolysis on cancer stem cell functions. Biochimie.

[41] Gopinath S., Malla R., Alapati K., Gorantla B., Gujrati M., Dinh D.H., Rao J.S. (2013). Cathepsin B and uPAR regulate self-renewal of glioma-initiating cells through GLI-regulated Sox2 and Bmi1 expression. Carcinogenesis.

[42] Moon E.K., Chung D.I., Hong Y.C., Kong H.H. (2007). Differentially expressed genes of *Acanthamoeba castellanii* during encystation. Korean J. Parasitol..

[43] Moon E.K., Chung D.I., Hong Y.C., Kong H.H. (2008). Characterization of a serine proteinase mediating encystation of *Acanthamoeba*. Eukaryot. Cell.

[44] Moon E.K., Chung D.I., Hong Y.C., Ahn T.I., Kong H.H. (2008). *Acanthamoeba castellanii*: Gene profile of encystation by ESTs analysis and KOG assignment. Exp. Parasitol..

[45] Dudley R., Alsam S., Khan N.A. (2008). The role of proteases in the differentiation of *Acanthamoeba castellanii*. FEMS Microbiol. Lett..

[46] Achar S.B., Weisman R.A. (1980). Adenylate cyclase activity during growth and encystment of *Acanthamoeba castellanii*. Biochim. Biophys. Acta.

[47] Hax W.M., van Venrooij G.E., Vossenberg J.B. (1974). Cell communication: A cyclic-AMP mediated phenomenon. J. Membr. Biol..

[48] Moon E.K., Chung D.I., Hong Y., Kong H.H. (2011). Atg3-mediated lipidation of Atg8 is involved in encystation of *Acanthamoeba*. Korean J. Parasitol..

[49] Mortazavi P.N., Keisary E., Loh L.N., Jung S.Y., Khan N.A. (2011). Possible roles of phospholipase A2 in the biological activities of *Acanthamoeba castellanii* (T4 genotype). Protist.

[50] Bennett D.T., Deng X.S., Jessica A.Y., Bell M.T., Mauchley D.C., Meng X., Reece T.B., Fullerton D.A., Weyan M.J. (2014). Cancer stem cell phenotype is supported by secretory phospholipase A2 in human lung cancer cells. Ann. Thorac. Surg..

[51] Malumbres M., Barbacid M. (2003). RAS oncogenes: The first 30 years. Nat. Rev. Can..

[52] Kobayashi A., Okuda H., Xing F., Pandey P.R., Watabe M., Hirota S., Pai S.K., Liu W., Fukuda K., Chambers C. (2011). Bone morphogenetic protein 7 in dormancy and metastasis of prostate cancer stem-like cells in bone. J. Exp. Med..

[53] Samuels Y., Wang Z., Bardelli A., Silliman N., Ptak J., Szabo S., Yan H., Gazdar A., Powell S.M., Riggins G.J. (2004). High Frequency of Mutations of the PIK3CA Gene in Human Cancers. Science.

[54] Clarke M., Lohan A.J., Liu B., Lagkouvardos I., Roy S., Zafar N., Bertelli C., Schilde C., Kianianmomeni A., Bürglin T.R. (2013). Genome of *Acanthamoeba castellanii* highlights extensive lateral gene transfer and early evolution of tyrosine kinase signaling. Gen. Biol..

[55] Siddiqui R., Jarroll E.L., Khan N.A. (2010). Balamuthia mandrillaris: Role of galactose in encystment and identification of potential inhibitory targets. Exp. Parasitol..

[56] Seo E.J., Kim D.K., Jang I.H., Choi E.J., Shin S.H., Lee S.I., Kwon S.M., Kim K.H., Suh D.S., Kim J.H. (2016). Hypoxia-NOTCH1-SOX2 signaling is important for maintaining cancer stem cells in ovarian cancer. Oncotarget.

[57] Agliano A., Calvo A., Box C. (2017). The challenge of targeting cancer stem cells to halt metastasis. Semin. Cancer Biol..

[58] Morrissey C., Vessella R.L., Lange P.H., Lam H.M. (2016). The biology and clinical implications of prostate cancer dormancy and metastasis. J. Mol. Med..

[59] Maruta H., Goldstein L. (1975). The fate and origin of the nuclear envelope during and after mitosis in Amoeba proteus. I. Synthesis and behavior of phospholipids of the nuclear envelope during the cell life cycle. J. Cell Biol..

[60] Neff R.J., Neff R.H. (1969). The biochemistry of amoebic encystment. Symp. Soc. Exp. Biol..

[61] Band R.N., Mohrlok S. (1973). The cell cycle and induced amitosis in *Acanthamoeba*. J. Protozool..

[62] Jantzen H., Schulze I., Stohr M. (1988). Relationship between the timing of DNA replication and the developmental competence in *Acanthamoeba castellanii*. J. Cell Sci..

[63] Stöhr M., Bommert K., Schulze I., Jantzen H. (1987). The cell cycle and its relationship to development in *Acanthamoeba castellanii*. J. Cell Sci..

[64] Mengue L., Régnacq M., Aucher W., Portier E., Héchard Y., Samba-Louaka A. (2016). Legionella pneumophila prevents proliferation of its natural host *Acanthamoeba castellanii*. Sci. Rep..

[65] Wong R.S.Y. (2011). Apoptosis in cancer: From pathogenesis to treatment. J. Exp. Clin. Cancer Res..

[66] Reed J.C. (1997). Bcl-2 family proteins: Regulators of apoptosis and chemoresistance in hematologic malignancies. Semin. Hematol..

[67] Selivanova G. (2004). p53: Fighting cancer. Curr. Cancer Drug Targets.

[68] Baig A.M., Lalani S., Khan N.A. (2017). Apoptosis in *Acanthamoeba castellanii* belonging to the T4 genotype. J. Basic Microbiol..

[69] Martín-Navarro C.M., López-Arencibia A., Sifaoui I., Reyes-Battle M., Valladares B., Martínez-Carretero E., Piñero J.E., Maciver S.K., Lorenzo-Morales J. (2015). Statins and voriconazole induce Programmed Cell Death in *Acanthamoeba*. Antimicrob. Agents Chemother..

[70] Sifaoui I., López-Arencibia A., Martín-Navarro C.M., Reyes-Batlle M., Wagner C., Chiboub O., Mejri M., Valladares B., Abderrabba M., Pinero J.E. (2017). Programmed cell death in *Acanthamoeba castellanii* Neff induced by several molecules present in olive leaf extracts. PLoS ONE.

[71] Yuan J., Yankner B.A. (2000). Apoptosis in the nervous system. Nature.

[72] Meslin B., Barnadas C., Boni V., Latour C., De Monbrison F., Kaiser K., Picot S. (2007). Features of apoptosis in Plasmodium falciparum erythrocytic stage through a putative role of PfMCA1 metacaspase-like protein. J. Inf. Dis..

[73] Kosec G., Alvarez V.E., Agüero F., Sánchez D., Dolinar M., Turk B., Turk V., Cazzulo J.J. (2006). Metacaspases of Trypanosoma cruzi: Possible candidates for programmed cell death mediators. Mol. Biochem. Parasitol..

[74] Feng Y., Hsiao Y.H., Chen H.L., Chu C., Tang P., Chiu C.H. (2009). Apoptosis-like cell death induced by Salmonella in *Acanthamoeba* rhysodes. Genomics.

[75] Nakisah M.A., Muryany M.I., Fatimah H., Fadilah R.N., Zalilawati M.R., Khamsah S., Habsah M. (2012). Anti-amoebic properties of a Malaysian marine sponge Aaptos sp. on *Acanthamoeba castellanii*. World J. Microbiol. Biotechnol..

[76] Wu D., Qiao K., Feng M., Fu Y., Cai J., Deng Y., Tachibana H., Cheng X. (2018). Apoptosis of Acanthamoeba castellanii trophozoites induced by oleic acid. J. Eukaryot. Microbiol..

[77] Semba T., Sammons R., Wang X., Xie X., Dalby K.N., Ueno N.T. (2020). JNK Signaling in Stem Cell Self-Renewal and Differentiation. Int. J. Mol. Sci..

[78] Weisman R.A. (1976). Differentiation in *Acanthamoeba castellanii*. Ann. Rev. Microbiol..

[79] Mehdi H., Garg N.K. (1987). Changes in the lipid composition and activities of isocitrate dehydrogenase and isocitrate lyase during encystation of *Acanthamoeba* culbertsoni strain A-1. Trans. R Soc. Trop. Med. Hyg..

[80] Hugo E.R., Byers T.J. (1993). S-adenosyl-L-methionine decarboxylase of *Acanthamoeba castellanii* (Neff): Purification and properties. Biochem. J..

[81] Bouyer S., Rodier M.H., Guillot A., Héchard Y. (2009). *Acanthamoeba castellanii*: Proteins involved in actin dynamics, glycolysis, and proteolysis are regulated during encystation. Exp. Parasitol..

[82] Rosen J.M., Jordan C.T. (2009). The increasing complexity of the cancer stem cell paradigm. Science.

[83] Heddleston J.M., Li Z., McLendon R.E., Hjelmeland A.B., Rich J.N. (2009). The hypoxic microenvironment maintains glioblastoma stem cells and promotes reprogramming towards a cancer stem cell phenotype. Cell Cycle.

[84] Gilkes D.M., Semenza G.L., Wirtz D. (2014). Hypoxia and the extracellular matrix: Drivers of tumour metastasis. Nat. Rev. Cancer.

[85] Bruttel V.S., Wischhusen J. (2014). Cancer stem cell immunology: Key to understanding tumorigenesis and tumor immune escape?. Front. Immunol..

[86] Brown T.J., Cursons R.T., Keys E.A. (1982). Amoebae from antarctic soil and water. Appl. Environ. Microbiol..

[87] Sawyer T.K., Visvesvara G.S., Harke B.A. (1977). Pathogenic amoebas from brackish and ocean sediments, with a description of *Acanthamoeba* hatchetti, n. sp.. Science.

[88] Davis P.G., Caron D.A., McN J. (1978). Oceanic amoebae from the North Atlantic: Culture, distribution, and taxonomy. Trans. Am. Microscop. Soc..

[89] Leger M.M., Gawryluk R.M., Gray M.W., Roger A.J. (2013). Evidence for a hydrogenosomal-type anaerobic ATP generation pathway in *Acanthamoeba castellanii*. PLoS ONE.

[90] Bowers B., Korn E.D. (1969). The fine structure of Acanthamoeba castellanii (Neff Strain): II. Encystment. J. Cell Biol..

[91] Hu Y., Fu L.W. (2012). Targeting cancer stem cells: A new therapy to cure cancer patients. Am. J. Cancer Res..

[92] Osman A., Afify S.M., Hassan G., Fu X., Seno A., Seno M. (2020). Revisiting Cancer Stem Cells as the Origin of Cancer-Associated Cells in the Tumor Microenvironment: A Hypothetical View from the Potential of iPSCs. Cancers.

[93] Kim Y.J., Siegler E.L., Siriwon N., Wang P. (2016). Therapeutic strategies for targeting cancer stem cells. J. Cancer Met. Treat.

[94] Lorenzo-Morales J., Khan N.A., Walochnik J. (2015). An update on Acanthamoeba keratitis: Diagnosis, pathogenesis and treatment. Parasite.

[95] Siddiqui R., Khan N.A. (2012). Biology and pathogenesis of Acanthamoeba. Parasites Vectors.

[96] Sekine R., Kawata T., Muramoto T. (2018). CRISPR/Cas9 mediated targeting of multiple genes in Dictyostelium. Sci. Rep..

[97] Lorenzo-Morales J., Martín-Navarro C.M., López-Arencibia A., Santana-Morales M.A., Afonso-Lehmann R.N., Maciver S.K., Valladares B., Martínez-Carretero E. (2010). Therapeutic potential of a combination of two gene-specific small interfering RNAs against clinical strains of Acanthamoeba. Antimicrob. Agents Chemother..

[98] Baig A.M., Khan N.A., Abbas F. (2015). Eukaryotic cell encystation and cancer cell dormancy: Is a greater devil veiled in the details of a lesser evil?. Can. Biol. Med..

